# Extended Real-World Efficacy of Faricimab in Therapy-Resistant Macular Edema Due to Retinal Vein Occlusion: 9-Month Follow-Up Results

**DOI:** 10.3390/jcm14207197

**Published:** 2025-10-13

**Authors:** Michael Hafner, Tina R. Herold, Alexander Kufner, Franziska Eckardt, Ben Asani, Siegfried G. Priglinger, Johannes Schiefelbein

**Affiliations:** Department of Ophthalmology, LMU University Hospital, LMU Munich, 81377 Munich, Germany

**Keywords:** faricimab, retinal vein occlusion, macular edema, VEGF, angiopoietin-2, intravitreal therapy, optical coherence tomography

## Abstract

**Background:** Macular edema (ME) secondary to retinal vein occlusion (RVO) is a significant cause of vision impairment. Many patients show suboptimal responses to anti-vascular endothelial growth factor (anti-VEGF) monotherapy, prompting the exploration of alternative treatments. Faricimab is a bispecific antibody that targets VEGF-A and angiopoietin-2. We report 9-month real-world outcomes of switching to faricimab in therapy-resistant RVO-associated ME. **Methods:** In this retrospective study at a single tertiary center, patients with persistent or recurrent ME despite prior treatments (ranibizumab, aflibercept, or dexamethasone implant) were switched to faricimab. All eyes received a loading phase of four monthly faricimab injections, followed by a treat-and-extend regimen individualized per response. Key outcomes included best-corrected visual acuity (BCVA, logMAR), the central subfield thickness (CST, μm), and the intraretinal fluid (IRF) status on optical coherence tomography, assessed from the baseline (month 0, mo0) through the loading phase (mo1–mo3) and at month 9 (mo9). **Results:** Nineteen eyes (19 patients, mean age 64.8 years) were analyzed. The median BCVA improved from 0.20 to 0.00 logMAR by month 3 (*p* < 0.01) and was maintained at month 9. The median CST decreased from 325 μm at the baseline to 285 μm at month 3 (*p* < 0.01) and remained at 285 μm at month 9. IRF was present in 100% of eyes at the baseline, 26% at month 3, and 26% at month 9 (*p* < 0.01 for the baseline vs. month 9). Among eyes previously on anti-VEGF therapy (*n* = 14), the median treatment interval increased from 45.50 days at the baseline to 56.50 days at month 9 (*p* = 0.01; δ = 0.86). No intraocular inflammation or other adverse events were observed in this cohort over nine months. **Conclusions:** In this retrospective series, switching to faricimab was associated with improvements in vision and retinal anatomy that were maintained over 9 months; injection intervals were extended in a subset of eyes. These exploratory findings warrant confirmation in larger, controlled studies to define long-term effectiveness, safety, and dosing strategies.

## 1. Introduction

Macular edema (ME) resulting from retinal vein occlusion (RVO) is a common, sight-threatening condition that impairs vision [1,2]. ME in RVO arises from disrupted retinal circulation and increased vascular permeability, leading to fluid accumulation in the macula and associated vision loss [3].

While intravitreal anti-vascular endothelial growth factor (anti-VEGF) agents have become an established therapy for RVO-related ME by counteracting VEGF-driven vascular leakage [4], real-world outcomes often fall short of those in clinical trials [5]. Factors such as variable patient adherence, the chronic disease course, and an incomplete response to therapy can leave a subset of patients with persistent or recurrent edema despite regular anti-VEGF injections. Additionally, sustained corticosteroid therapy (e.g., dexamethasone implants) can effectively reduce inflammation and edema in RVO; however, its use is limited by side effects, including the elevation of intraocular pressure, cataract formation, and implant migration [6,7]. Thus, treatments are needed that address refractory ME and may reduce the injection burden.

Emerging evidence suggests that multiple pathways contribute to RVO-related ME. Besides VEGF, elevated levels of angiopoietin-2 (Ang-2) contribute to disease progression by destabilizing retinal vasculature [8]. Ang-2 antagonizes the Tie2 receptor signaling (usually maintained by Ang-1), leading to increased vascular permeability and inflammation [9]. This suggests that blocking VEGF alone may be insufficient in some cases of ME; a combined inhibition of VEGF and Ang-2 has been hypothesized to modulate vascular stability [10]. Faricimab (Vabysmo^®^, Roche/Genentech, South San Francisco, CA, USA) inhibits VEGF-A and Ang-2 [11], two pathways implicated in RVO-related ME [12].

Phase III trials in DME [13] and nAMD [14] showed visual outcomes non-inferior to aflibercept. In RVO (BALATON/COMINO), 24-week outcomes were comparable to aflibercept [12] and 72-week data suggest that extended treat-and-extend schedules are feasible in a proportion of patients [15].

Translating these results to real-world settings, particularly in pretreated, therapy-resistant cohorts, remains important [16]; early reports indicate a potential benefit, though evidence is still limited. For example, short-term outcomes in treatment-naïve versus switched RVO patients treated with faricimab on a pro re nata regimen demonstrated a central thickness reduction in both groups, with vision improvement, especially in treatment-naïve eyes [17]. Another report on patients with chronic RVO ME refractory to aflibercept found that switching to faricimab led to rapid anatomical and functional improvements, allowing injection intervals to be lengthened from 12 weeks to 16 weeks over a six-month period [18]. These reports describe CST reductions and, in some cohorts, longer dosing intervals; generalizability remains limited.

We previously reported short-term improvements after a 3-month loading phase of faricimab [19]. The present study extends prior observations by examining whether the visual and anatomical changes seen at 3 months persisted to 9 months and by describing post-switch injection interval changes in a pretreated cohort.

## 2. Materials and Methods

### 2.1. Study Design and Participants

This was a retrospective observational analysis of patients with RVO-related ME who were switched to faricimab after demonstrating resistance or poor response to prior therapies. Patient data were extracted from the Smart Eye database of the Department of Ophthalmology, LMU University Hospital Munich, from July 2024 to August 2025. All consecutive eligible eyes were considered for switching and inclusion; exclusions were limited to the prespecified criteria below. The decision to switch was made by the treating physician in discussion with the patient based on disease activity and safety considerations.

Inclusion criteria were (i) a diagnosis of branch RVO (BRVO) or central/hemiretinal RVO (CRVO) with center-involving ME. (ii) An inadequate anatomical or functional response to previous standard treatments, defined as persistent or recurrent intraretinal fluid on optical coherence tomography (OCT) despite consistent therapy. Eyes previously treated with ranibizumab or aflibercept were defined as treatment-resistant when IRF remained despite three consecutive monthly injections, or when any attempt to extend the injection interval beyond six weeks led to a recurrence of edema. In cases pretreated with a dexamethasone implant (Ozurdex^®^, Allergan Inc., Irvine, CA, USA), resistance was characterized by either persistent or relapsing ME despite adequate steroid exposure, or by an early recurrence of fluid within three months, preceding the expected pharmacologic duration of action. Eyes showing steroid-related complications, such as a marked intraocular pressure increase above 25 mmHg or anterior chamber implant displacement [6,7], were likewise transitioned to faricimab for safety reasons. (iii) All patients included had undergone at least a 9-month follow-up after switching, with a loading phase of four approximately monthly faricimab injections within a period of 3 ± 1 months and subsequent visits up to mo9. Exclusion criteria included any other ocular pathology that could independently cause ME or affect vision, such as diabetic macular edema, vitreomacular traction, uveitis, retinal surgery within the past 6 months, or uncontrolled glaucoma (IOP > 30 mmHg).

This study adhered to the principles outlined in the Declaration of Helsinki. It was reviewed by the Institutional Ethics Board, which granted a waiver of formal approval (LMU Munich ID: 25-0070-KB) due to the retrospective design and use of de-identified data.

### 2.2. Treatment Protocol

The initial treatment phase consisted of four intravitreal injections of faricimab (6.0 mg) administered at roughly monthly intervals (minimum spacing of 28 days) during the first three (±1) months after baseline. After the loading phase (months 0–3), patients were managed with an individualized treat-and-extend regimen. If clinical signs were stable or improved, intervals were extended by 2–4 weeks beyond 4 weeks; they were shortened if activity recurred. By mo9, each eye had reached a personalized maintenance interval or was still under adjustment based on ongoing response.

Each injection was carried out under sterile conditions following the institution’s routine intravitreal protocol. Patients continued to receive ancillary RVO management as needed; notably, a subset with extensive non-perfusion had previously undergone retinal laser photocoagulation to reduce ischemia-driven VEGF production (10 patients in the overall cohort). No new laser treatments were administered after the switch to faricimab during the observation period.

### 2.3. Assessments and Data Collection

Baseline data collected included patient demographics (age, sex), RVO type (branch vs. central/hemiretinal), duration of ME, and a detailed treatment history (number of prior intravitreal anti-VEGF injections and Ozurdex implants, prior retinal laser treatments, etc.). Before treatment, BCVA was assessed, beginning with objective autorefraction (Nidek AR-1s), followed by subjective refinement using a 4 m Snellen chart. IOP was measured using non-contact tonometry (Nidek^®^ REF/KERATO/TONOMETER Tonoref II, Nidek Co., Ltd., Gamagori, Japan), followed by a dilated fundus examination. Evaluation of macular structure and retinal fluid was routinely conducted with SD-OCT and near-infrared imaging on the Spectralis HRA + OCT platform (Heidelberg Engineering, Heidelberg, Germany). RVO diagnosis was first made using OCT in combination with ultrawide-field fluorescein angiography. Quantitative OCT metrics, such as CST and retinal volume, were extracted via Heidelberg Eye Explorer (v1.10.12.0), with manual corrections applied when required, and referenced to ETDRS grid sectors [20].

Follow-up visits occurred monthly during the loading phase (months 1, 2, and 3) and then according to the treat-and-extend schedule. For analysis, we focused on outcomes at the end of loading (mo3) and at the final 9-month follow-up (mo9), comparing them to baseline (mo0).

### 2.4. Data Management and Statistical Analysis

All data were recorded in Microsoft Excel (Version 16.78.3 for Mac) and analyzed using GraphPad Prism 10 (GraphPad Software, San Diego, CA, USA; Version 10.3.1 for macOS) or, in cases of more complex testing, R (R Foundation for Statistical Computing, Vienna, Austria) within RStudio 2024.12.1 + 563 (R version 4.5.0). Continuous variables are reported as median with interquartile range (IQR) or mean ± standard deviation, as appropriate; categorical variables (e.g., proportion of eyes with IRF) are given as percentages ± standard error of the mean (SEM). Shapiro–Wilk assessed normality. As most outcomes were non-normal, non-parametric methods were used. For repeated measures compared across three or more time points, we applied the Friedman test; when significant, Dunn’s multiple comparisons test was used for post hoc pairwise contrasts. When only two time points were directly compared (e.g., baseline vs. month 9), the Wilcoxon signed-rank test was used. For categorical longitudinal data (IRF presence across time points), we used Cochran’s Q test, followed by post hoc McNemar tests with Holm correction across all pairwise contrasts. A *p* < 0.05 was considered statistically significant. Effect sizes for paired non-parametric comparisons were quantified using Cliff’s delta (δ) to contextualize the magnitude of change (notably for interval extension).

## 3. Results

### 3.1. Baseline Demographics

Nineteen eyes from 19 patients met the inclusion criteria, including 11 males and eight females with a mean age of 64.8 ± 12.7 years. The cohort represents consecutive eligible cases within this study’s time frame. The mean follow-up period was 8.9 ± 1.3 months. Before the switch, patients had a prolonged and intensive treatment history. Before faricimab, eyes had an average of 33.2 ± 26.1 treatments, including 16.2 ranibizumab and 15.6 aflibercept injections, and 1.4 Ozurdex implants per eye (some eyes received multiple therapies sequentially). The annual injection rate before switch was 9.3 ± 3.0 injections, reflecting a high treatment burden in this cohort. Immediately before switching, nine patients were on ranibizumab, five on aflibercept, and five had most recently received an Ozurdex^®^ implant. Detailed data are provided in Table 1. No patient underwent cataract surgery during the follow-up period.

As part of their earlier treatment history, 10 patients in our cohort had undergone retinal laser treatment using either the PASCAL (Optimedica Corp., Santa Clara, CA, USA) or Navilas 577s (OD-OS GmbH, Teltow, Germany) systems. Sectoral laser treatment was guided by ultrawide-field fluorescein angiography to target ischemic retinal areas following RVO, with the intention of reducing VEGF-driven activity and potentially decreasing the need for injections [21]. This illustrates the therapeutic complexity of our cohort and the limitations of anti-VEGF or corticosteroid monotherapy in certain patients. Importantly, all laser procedures had been completed at least three months prior to the switch to faricimab, and no additional laser treatments were performed thereafter or during the follow-up period. A direct confounding effect on post-switch outcomes is, therefore, unlikely.

Fourteen of the 19 eyes had been on anti-VEGF therapy immediately before switching (five others had last received steroids). As prior steroid exposure could confound some outcomes (e.g., those eyes might have been changed due to an IOP rise rather than pure resistance), we also analyzed this anti-VEGF-pretreated subgroup separately. Their baseline profile (Table 2) was similar. They had received, on average, 29.0 ± 23.9 anti-VEGF injections before switching to a slightly higher annual frequency (10.2 injections/year). This subgroup’s prior treatment interval before faricimab was a median of 45.50 days, reflecting the shortest cycle that maintained stability with previous anti-VEGF (often a treat-and-extend plateau they could not extend beyond). This serves as a baseline for evaluating changes in the post-switch interval.

A total of five eyes (*n* = 5) had received intravitreal steroid therapy immediately before switching to faricimab. The mean age of these patients was 64.2 ± 9.9 years, comprising four males and one female. The mean number of prior intravitreal injections before switching was 45.0 ± 31.2, including both anti-VEGF (ranibizumab: 3.0 ± 4.2; aflibercept: 37.2 ± 36.4) and steroid treatments (Ozurdex: 4.8 ± 5.6). Under steroid therapy, the final median steroid-specific interval immediately prior to switching was 112 (IQR: 28) days, with a mean of 6.6 ± 1.4 injections (steroid and anti-VEGF) per year (mean duration of RVO before switch: 6.7 ± 3.8 years). Four of these patients had undergone retinal laser coagulation prior to the switch.

### 3.2. Visual Acuity (BCVA)

**Figure 1:**
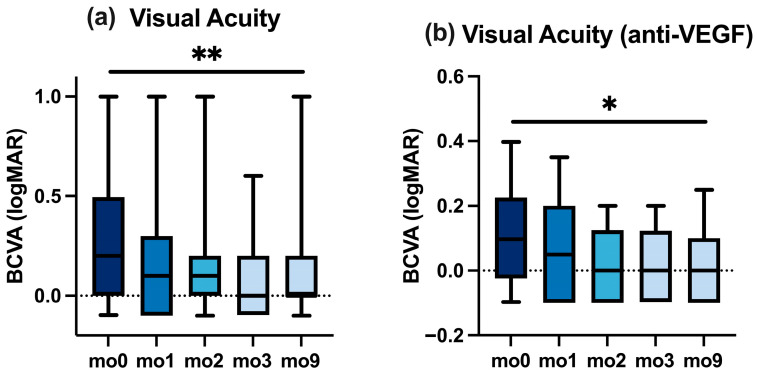
Best-corrected visual acuity (BCVA, logMAR) at baseline (mo0) and follow-up (mo1–mo9). (**a**) Overall cohort. (**b**) Anti-VEGF-pretreated subgroup. Boxplots with whiskers; * *p* < 0.05, ** *p* < 0.01.

Figure 1a illustrates the BCVA trajectory for the overall cohort (including patients switched from anti-VEGF agents as well as Ozurdex^®^). Figure 1b illustrates the BCVA trajectory for the subgroup treated with an anti-VEGF agent before the switch.

After the switch, median BCVA changed from 0.20 logMAR at mo0 to 0.10 at mo1 and 0.00 at mo3, and remained 0.00 at mo9 (overall *p* < 0.01). Corresponding data are summarized in Figure 1a and Table 3.

The anti-VEGF-pretreated subgroup followed a similar BCVA trajectory as the overall cohort; we, therefore, summarize without repeating numbers (see Figure 1b and Table 4).

### 3.3. OCT Anatomical Outcomes

**Figure 2:**
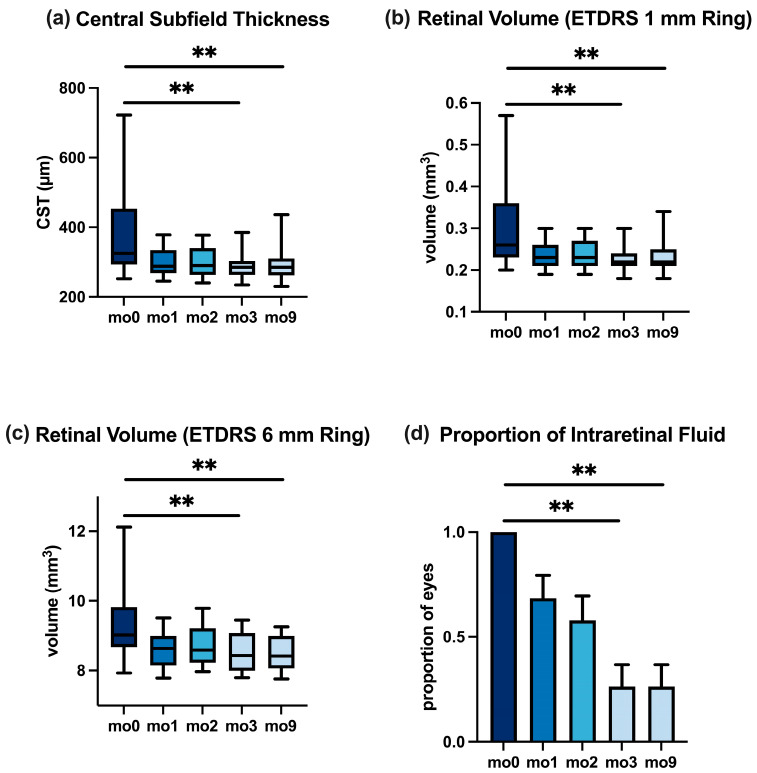
Changes in OCT biomarkers in the overall cohort from baseline (mo0) to month 9. (**a**) Central subfield thickness (CST). (**b**,**c**) Retinal volume in ETDRS 1 mm and 6 mm grids. (**d**) Proportion of eyes exhibiting IRF. Boxplots (**a**–**c**), bar plot (**d**); ** *p* < 0.01.

**Central Subfield Thickness.** The median CST was 325 μm at the baseline, reflecting macular thickening. After one faricimab injection (mo1), the CST fell to 288 μm, and by mo3, it further reduced to 285 μm (*p* < 0.01 for mo0 vs. mo3). At mo9, the median CST was 285 μm, essentially unchanged from mo3 (*p* > 0.99 for mo3 vs. mo9), indicating that the anatomical improvement achieved after the loading phase was maintained through the extended follow-up. Figure 2a demonstrates the CST over time; no rebound edema was observed from mo3 to mo9. Overall, the CST at mo9 remained lower than the baseline (*p* < 0.01 for mo0 vs. mo9).

**Figure 3:**
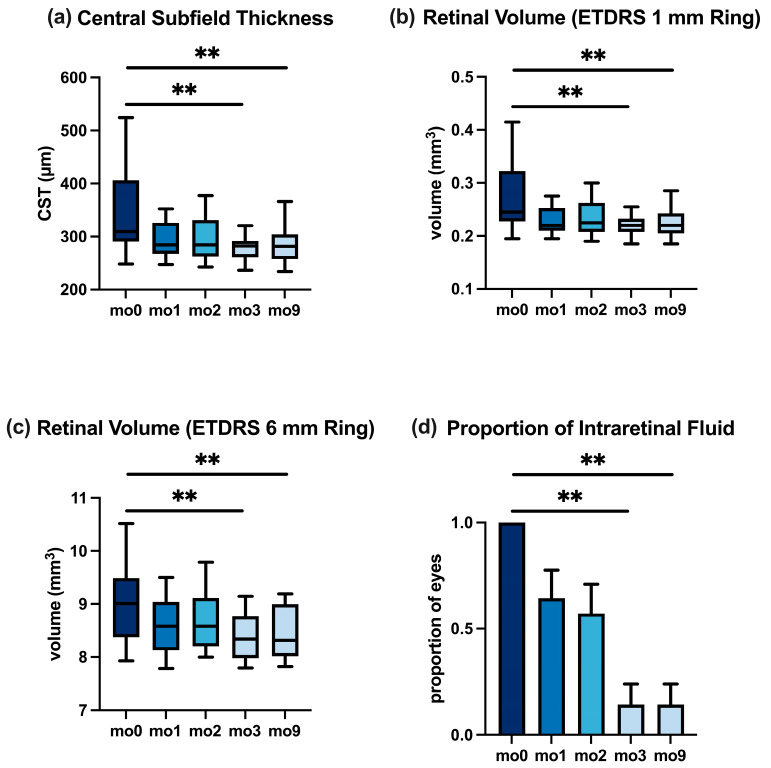
Changes in OCT biomarkers in the anti-VEGF-pretreated subgroup from baseline (mo0) to month 9. (**a**) CST. (**b**,**c**) Retinal volume in ETDRS 1 mm and 6 mm grids. (**d**) Proportion of eyes exhibiting IRF. Boxplots (**a**–**c**), bar plot (**d**); ** *p* < 0.01.

In the anti-VEGF-pretreated subgroup, the CST changes mirrored the overall pattern; detailed values are reported in Table 4 and Figure 3.

**Retinal Volume.** Changes in retinal thickening were also reflected in the retinal volume measurements. Within the central 1 mm ETDRS circle, the median volume reduced from 0.26 mm^3^ at mo0 to 0.22 mm^3^ at mo3 (*p* < 0.01) and stayed at 0.22 mm^3^ at mo9. Similarly, in the 6 mm macular grid, the median volume dropped from 9.0 mm^3^ at the baseline to 8.4 mm^3^ at mo3 (*p* < 0.01) and remained 8.4 mm^3^ at mo9. These sustained volume reductions support the findings of the CST (Table 3). Figure 2b,c illustrate these volume trends for the 1 mm and 6 mm zones, respectively. In the anti-VEGF subgroup, the pattern was similar (Table 4), illustrated in Figure 3b,c.

**Intraretinal Fluid.** The proportion of eyes with intraretinal fluid decreased from 100% at the baseline to 26% at mo3 and was maintained at 26% at mo9 on extended intervals (*p* < 0.01 for mo0 vs. mo9). The values are shown in Figure 2d and Table 3.

The reduction in intraretinal fluid for the anti-VEGF-pretreated cohort paralleled the overall cohort (Figure 3; Table 4).

**Figure 4:**
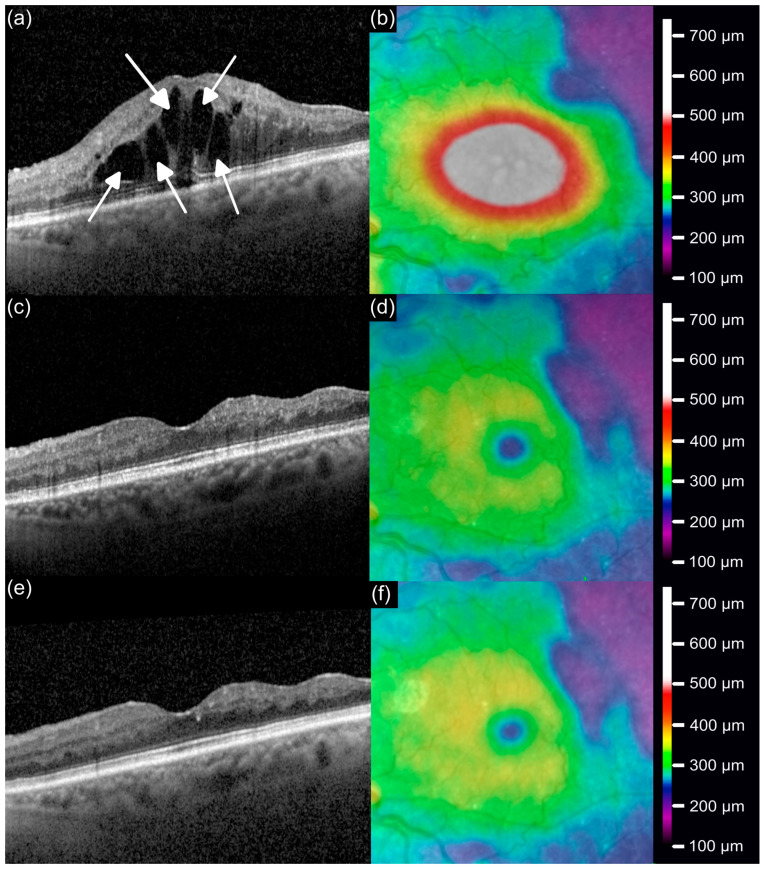
Representative BRVO case switched from ranibizumab to faricimab. OCT and thickness maps at (**a**,**b**) baseline, (**c**,**d**) month 3, and (**e**,**f**) month 9. Arrows indicate IRF.

**Illustrative Case.** Figure 4 illustrates a representative patient (69-year-old female with BRVO) who had previously been treated with ranibizumab every 42 days, but continued to exhibit extensive cystoid IRF and reduced vision (0.5 logMAR) (Figure 4a,b). After switching to faricimab, the interval was initially shortened to 28 days during the loading phase, resulting in a reduction of retinal fluid and restoration of the foveal architecture, accompanied by visual recovery to 0.0 logMAR at mo3 (Figure 4c,d). Then, the interval was successfully re-extended to 42 days and at mo9, the macula remained structurally stable with a sustained functional improvement (0.0 logMAR; Figure 4e,f).

### 3.4. Safety and Intraocular Pressure

No intraocular inflammation or other adverse events were recorded in this cohort over 9 months; this study was not powered to detect rare events. The intraocular pressure remained stable. The median IOP was 16 mmHg at the baseline and at mo3 and mo9, with no significant change (*p* = 0.81).

### 3.5. Treatment Interval Extension

One of the goals of switching to faricimab was to reduce the treatment. After the 3-month loading phase, clinicians began extending the injection intervals for stable patients. By the 9-month visit, extension decisions resulted in a range of maintenance intervals across patients, individualized by their disease activity.

**Figure 5:**
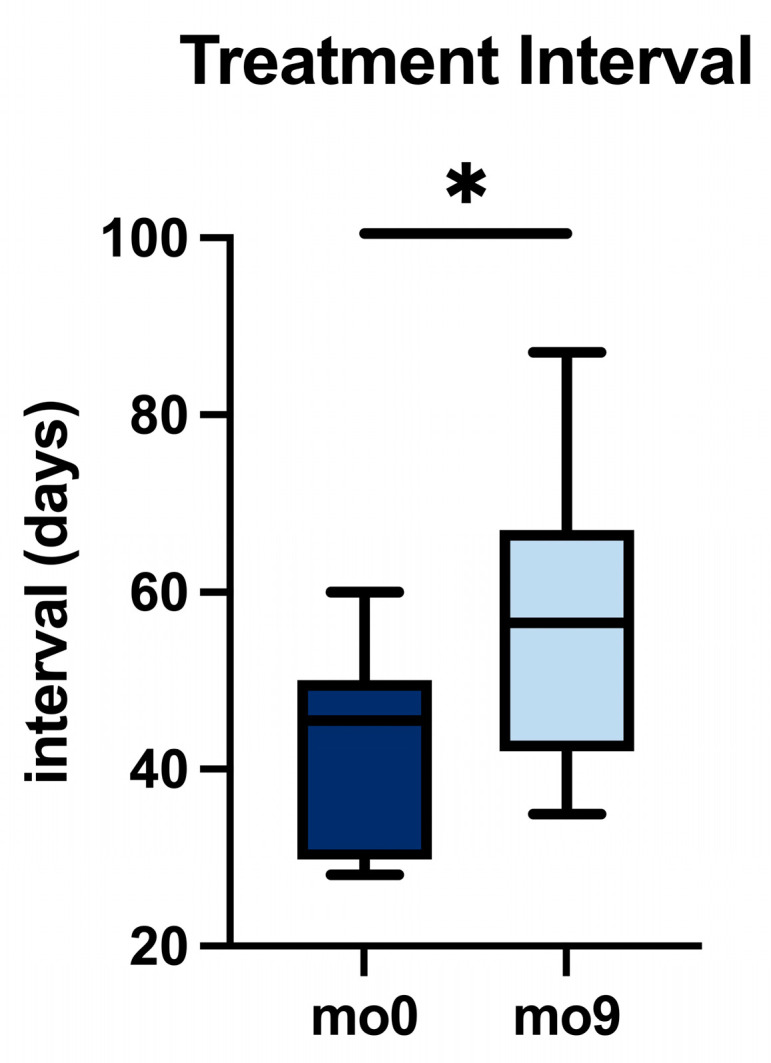
Change in treatment interval in anti-VEGF-pretreated eyes from baseline (mo0) to month 9 (mo9). Boxplots; * *p* < 0.05.

In anti-VEGF-pretreated eyes (*n* = 14), the median injection interval increased from 45.5 to 56.5 days by month 9 (*p* = 0.01; δ = 0.86). Figure 5 charts the distribution of intervals pre- and post-switch.

The distribution of extended intervals (anti-VEGF pre-treated eyes) was as follows: 1 eye (7.1%) in the 4–6-week range, five eyes (35.7%) in the 6–8-week range, five eyes (35.7%) in the 8–10-week range, one eye (7.1%) in the 10–12-week range, and two eyes (14.3%) beyond 12 weeks. No eyes had yet reached 14 or 16 weeks within the available follow-up period. The values are given in Table 5.

## 4. Discussion

To the best of our knowledge, this is the first real-world study to evaluate the longer-term outcomes (≥9 months) of faricimab in refractory RVO cases. Improvements observed after the loading phase were maintained to month 9 under interval extension. The small sample size (*n* = 19) and lack of a concurrent control group mean that all efficacy signals are exploratory. This pattern is compatible with the dual-target mechanism of faricimab [15], but causal inference is not possible in this study design.

The baseline characteristics of our cohort highlight the challenges faced by this group. These eyes represented cases with an insufficient response to conventional monotherapy, and some had even undergone adjunct laser for ischemia without adequate control. After the switch, functional and anatomical measures remained stable over 9 months in this cohort. Dual-pathway inhibition (VEGF-A/Ang-2) has been proposed [14]; our findings are compatible with this but do not allow causal inference.

This aligns with other early real-world findings. For instance, Hirakata et al. reported CST reductions with a faricimab pro re nata (PRN, as needed) regimen in both naïve and anti-VEGF-switched eyes with RVO [17].

Noninfectious intraocular inflammation has been reported rarely after intravitreal faricimab, typically presenting as mild-to-moderate anterior/intermediate uveitis [22,23]. Notably, we observed no cases of intraocular inflammation/uveitis over 9 months in this cohort. As all laser treatments, part of the treatment history, were performed at least three months before the switch to faricimab and none during follow-up, we consider a direct confounding effect on the early treatment response unlikely.

In the anti-VEGF-pretreated subgroup, the median treatment interval increased by ~11 days to mo9. Before the switch, our patients were often on intensive regimens (some essentially on 4-to-6-weekly anti-VEGF indefinitely). We recognize that the extension was modest; however, even a 10–11-day increase can reduce annual injections by 2–3.

Other studies mirror this finding. Hikichi et al. reported that in RVO eyes refractory to aflibercept, faricimab allowed for an extension from an average of 12.3 weeks to 16.2 weeks over 6 months, alongside improvements in CST and vision [18]. Our extension was more moderate than in some reports; differences in populations and protocols may contribute. Extended dosing has been reported in neovascular AMD and diabetic macular edema trials and real-world series [13,14,24].

It is worth discussing that not all eyes could be extended; a subset of about one-fifth of our cohort still required monthly injections to control edema. For such severe cases, combination approaches may be considered in the future (e.g., laser for nonperfusion or adjunctive steroid therapy). We observed that even in eyes with residual fluid, the amount was often less and vision remained stable. Consequently, some patients and clinicians opted to tolerate tiny cysts in exchange for longer intervals—a common real-world compromise. Under a treat-and-extend approach, intervals were individualized and varied across patients.

It is essential to contextualize our results in light of this study’s limitations. First, this retrospective, single-center study included a small cohort (*n* = 19) and lacked a concurrent control group, which limits both the statistical power and generalizability. Therefore, the results should be interpreted as hypothesis-generating. These outcomes should be interpreted cautiously. Larger studies are needed to define the range of responses better. Second, the 9-month follow-up is still relatively short for assessing long-term efficacy and safety. RVO is often a chronic condition, and whether faricimab’s benefits persist beyond one year remains to be seen. Nonetheless, our 9-month data provide an early look at mid-term outcomes in a real-world setting. Although we used consecutive inclusion, selection bias remains a possibility in a real-world setting because the decision to switch may be influenced by clinician judgment, patient preference, prior treatment tolerability (e.g., a steroid-related increase in intraocular pressure), and perceived disease activity. As a result, the cohort may over-represent eyes with greater activity or a higher likelihood of switching, which should be considered when interpreting the generalizability. There was no parallel group continuing on prior therapy for comparison, so we cannot attribute all gains solely to faricimab. Moreover, due to the retrospective design and incomplete documentation of systemic comorbidities in some patients, information on hypertension, diabetes, and dyslipidemia could not be consistently obtained and was, therefore, excluded to avoid reporting potentially biased or incomplete data. Our cohort presented with relatively high-baseline visual acuity potential, which may have contributed to the favorable outcomes observed. Therefore, these results should be interpreted with caution, as they may not be directly generalizable to patients with poorer baseline vision or a longer-standing disease. By design, a 9-month observation period does not permit a meaningful evaluation of 16-week durability. Confirming stability at 16 weeks would require well over one year of follow-up time. Accordingly, our findings should be interpreted as evidence of a favorable early-stage interval extension.

Despite these limitations, our study provides early real-world observations. Faricimab was introduced in Europe for RVO in July 2024 [25], and post-approval data have lagged behind clinical trials. Our findings add to this evidence base: in a pretreated cohort, dual Ang-2/VEGF inhibition was associated with anatomical and functional improvements and, in a subset of eyes, with longer injection intervals. Interpretation should remain cautious given the retrospective design and small sample size, and longer-term safety requires further assessment. Early nAMD reports suggest that extended intervals can reduce clinic visits and injections [24], but whether similar effects apply to RVO needs confirmation.

Open questions include whether baseline biomarkers (e.g., the extent of nonperfusion, inflammatory signs, hyper-reflective foci) help identify eyes more likely to benefit from Ang-2 inhibition and how far long-term safety in RVO populations can be characterized. Combination strategies in refractory cases (e.g., adjunctive laser or steroid) could be evaluated to optimize fluid control. Health–economic studies are warranted to determine whether any reduction in the injection frequency translates into cost or quality-of-life benefits.

In conclusion, these 9-month real-world data suggest that faricimab may represent a treatment option for ME secondary to RVO, particularly in eyes with an incomplete response to prior anti-VEGF or corticosteroid therapy. In this cohort, improvements observed after switching were maintained to month 9 and injection intervals were extended in a subset of eyes. These exploratory findings are compatible with a role for dual-pathway inhibition in retinal vascular disease, but do not establish comparative effectiveness. Prospective, controlled studies with a longer follow-up are needed to clarify long-term outcomes, safety, and optimal dosing.

## Figures and Tables

**Table 1:** Treatment history and baseline demographics of the study cohort (All Eyes, *n* = 19).

Number of participants	19
Number of eyes	19
Mean age (years)	64.8 ± 12.7
Gender	
Male	11
Female	8
Mean number of intravitreal treatments before switching to faricimab	
Total (n)	33.2 ± 26.1
Total Ranibizumab	16.2 ± 23.2
Total Aflibercept	15.6 ± 23.9
Total dexamethasone implants (Ozurdex®)	1.4 ± 3.4
Mean injections per year before switching to Faricimab	9.3 ± 3.0
Mean duration of RVO prior to switch to Faricimab (years)	4.1 ± 3.1
Last injection before switching to Faricimab	
Ranibizumab	9
Aflibercept	5
Dexamethasone implant (Ozurdex®)	5
Retinal laser coagulation	10

**Table 2:** Treatment history and baseline demographics of the sub-cohort treated with anti-VEGF medication (ranibizumab or aflibercept) right before the switch (*n* = 14).

Number of participants	14
Number of eyes	14
Mean age (years)	65.1 ± 13.9
Gender	
Male	7
Female	7
Mean number of intravitreal treatments before switching to faricimab	
Total (n)	29.0 ± 23.9
Total Ranibizumab	20.9 ± 25.5
Total Aflibercept	7.9 ± 11.7
Mean injections per year before switching to Faricimab	10.2 ± 2.9
Mean duration of RVO prior to switch to Faricimab (years)	3.2 ± 2.4
Last injection before switching to Faricimab	
Ranibizumab	9
Aflibercept	5
Retinal laser coagulation	6

**Table 3:** For the overall cohort, functional and anatomical parameters were assessed at baseline (mo0) and at follow-up visits (mo1, mo2, mo3, and mo9). The analyses included BCVA (logMAR), IOP (mmHg), CST (µm), retinal volumes in the central 1 mm and 6 mm ETDRS grids (mm^3^), and the proportion of eyes with IRF. Continuous variables are presented as median with IQR, while categorical data (IRF) are expressed as proportion ± SEM. *p*-values are reported for overall group comparisons and for specific intervals (mo0–mo3, mo3–mo9, and mo0–mo9). Statistically significant outcomes are highlighted in bold.

	BCVA (logMAR)	IOP (mmHg)	CST (µm)	Volume: ETDRS 1 mm (mm^3^)	Volume: ETDRS 6 mm (mm^3^)	IRF (Proportion of Eyes %)
month 0	0.20	16	325	0.26	9.00	100%
month 1	0.10	15	288	0.23	8.60	68%
month 2	0.10	16	290	0.23	8.60	58%
month 3	0.00	16	285	0.22	8.40	26%
month 9	0.00	16	285	0.22	8.40	26%
IQR (month 0)	0.50	3	160	0.13	1.10	SEM: 0%
IQR (month 1)	0.40	7	66	0.05	0.90	SEM: 11%
IQR (month 2)	0.20	7	77	0.06	1.00	SEM: 12%
IQR (month 3)	0.30	5	40	0.03	1.10	SEM: 10%
IQR (month 9)	0.20	6	48	0.04	0.90	SEM: 10%
*p*		0.81	**<0.01**	**<0.01**	**<0.01**	**<0.01**
*p* (mo0–mo3)			**<0.01**	**<0.01**	**<0.01**	**<0.01**
*p* (mo3–mo9)			>0.99	>0.99	>0.99	>0.99
*p* (mo0–mo9)	**<0.01**		**<0.01**	**<0.01**	**<0.01**	**<0.01**

**Table 4:** For the anti-VEGF-pretreated subgroup, both functional and anatomical parameters were examined at baseline (mo0) and at follow-up visits (mo1, mo2, mo3, and mo9). The analyses included BCVA (logMAR), IOP (mmHg), CST (µm), retinal volumes in the central 1 mm and 6 mm ETDRS grids (mm^3^), and the proportion of eyes with IRF. Continuous variables are presented as median with IQR, while categorical data (IRF) are expressed as proportion ± SEM. *p*-values are provided for overall comparisons and for specific intervals (mo0–mo3, mo3–mo9, and mo0–mo9). Statistically significant outcomes are emphasized in bold.

	BCVA (logMAR)	IOP (mmHg)	CST (µm)	Volume: ETDRS 1 mm (mm^3^)	Volume: ETDRS 6 mm (mm^3^)	IRF (Proportion of Eyes %)
month 0	0.10	16	310	0.25	9.01	100%
month 1	0.05	16	284	0.22	8.58	64%
month 2	0.10	16	284	0.23	8.58	57%
month 3	0.00	17	282	0.22	8.34	14%
month 9	0.00	16	282	0.22	8.32	14%
IQR (month 0)	0.25	4	116	0.10	1.12	SEM: 0%
IQR (month 1)	0.30	7	58	0.04	0.91	SEM: 13%
IQR (month 2)	0.22	6	69	0.06	0.91	SEM: 14%
IQR (month 3)	0.22	4	31	0.03	0.80	SEM: 10%
IQR (month 9)	0.20	6	47	0.04	0.98	SEM: 10%
*p*		0.81	**<0.01**	**<0.01**	**<0.01**	**<0.01**
*p* (mo0–mo3)			**<0.01**	**<0.01**	**<0.01**	**<0.01**
*p* (mo3–mo9)			>0.99	>0.99	>0.99	>0.99
*p* (mo0–mo9)	**0.02**		**<0.01**	**<0.01**	**<0.01**	**<0.01**

**Table 5:** Treatment interval changes in the anti-VEGF-pretreated subgroup at baseline (mo0) and month 9 (mo9). Data are presented as median with interquartile range (IQR). The *p*-value refers to the comparison between mo0 and mo9, with Cliff’s delta (δ) reported as a measure of effect size. Significant *p*-value shown in bold.

	Treatment Interval (Days)
mo0	45.50
mo9	56.50
IQR (mo0)	20.25
IQR (mo9)	25.00
*p*	**0.01**
δ	0.86

## Data Availability

Datasets supporting the findings of this study can be obtained from the corresponding author on reasonable request, provided that access complies with data privacy rules and the legal requirements in force in the respective jurisdiction.

## Abbreviations

Ang-2	Angiopoietin-2
anti-VEGF	Anti-vascular endothelial growth factor
BCVA	Best-corrected visual acuity
CST	Central subfield thickness
IOP	Intraocular Pressure
IQR	Interquartile range
IRF	Intraretinal fluid
nAMD	Neovascular age-related macular degeneration
OCT	Optical coherence tomography
PRN	Pro re nata (as needed)
RVO	Retinal vein occlusion
SEM	Standard error of the mean
